# Cystopexy raises the post-operative complication rate during laparoscopic hysterectomy for uterine prolapse

**DOI:** 10.3389/fsurg.2025.1488775

**Published:** 2025-04-30

**Authors:** Carlo Ronsini, Giada Andreoli, Marco Torella, Paola Romeo, Giuseppe Sarpietro, Stefano Cianci

**Affiliations:** ^1^ Department of Woman, Child and General and Specialized Surgery, University of Campania “Luigi Vanvitelli”, Naples, Italy; ^2^ Gynecology and Obstetrics Unit, Department of Human Pathology of Adult and Childhood “G. Barresi”, University of Messina, Messina, Italy; ^3^ Department of General Surgery and Medical Surgical Specialties, University of Catania, Catania, Italy

**Keywords:** pelvic organ prolapse, laparoscopic colposuspension, shull technique, natural vaginal tissue repair, cystopexy

## Abstract

**Objectives:**

This study aims to compare the outcomes of laparoscopic colposuspension sec Shull (LCSS) and laparoscopic colposacropexy (LCSP) with and without the addition of cystopexy for the treatment of pelvic organ prolapse (POP) in terms of postoperative complications, recurrence rates, and overall effectiveness.

**Materials and methods:**

A retrospective case-control analysis was conducted on women treated for grade 3–4 POP-Q uterine prolapse at the Academic Departments of Gynaecology and Obstetrics of “G. Martino” of Messina, Italy, and “L. Vanvitelli” of Napoli, Italy, between November 2020 and February 2022. Group A consisted of patients who underwent laparoscopic hysterectomy followed by LCSS or LCSP without cystopexy. At the same time, Group B included patients who had the same procedures with the addition of cystopexy. Data on complications were collected using the Clavien-Dindo classification, and prolapse recurrence was monitored according to the POP-Q system. Statistical analysis was performed using Fisher's exact, Chi-squared, and Wilcoxon rank-sum tests.

**Results:**

A total of 148 patients were included, with 125 in Group A and 23 in Group B. Group B showed a significantly higher rate of postoperative complications (16%) compared to Group A (2.4%) (*p* = 0.016), with an Odds Ratio of 7.62 (95% CI 1.59–36.51, *p* = 0.0017). No significant difference between the groups was found in the recurrence rate of prolapse at 24 months (*p* > 0.9).

**Conclusion:**

Adding cystopexy to LCSS or LCSP increases the risk of postoperative complications without reducing prolapse recurrence rates. Further research is needed to identify patients who may benefit from cystopexy and to evaluate its impact on stress incontinence and patient satisfaction.

## Highlights

Comparison of LCSS and LCSP with and without cystopexy for pelvic organ prolapse showed a significant increase in postoperative complications with cystopexy (16% vs. 2.4%). No significant difference was found in prolapse recurrence rates at 24 months.

## Introduction

Pelvic Organ Prolapse (POP) represents a significant health concern for women, affecting both their quality of life and daily functioning. It is estimated that 40% of women suffer from POP, particularly those over 50 years old, with a peak in incidence in women between 60 and 69 years old (1–3). Various non-surgical and surgical interventions have been developed to address POP and improve overall quality of life. Among the surgical options, the laparoscopic ones have gained prominence due to their minimally invasive nature, increased visualization of anatomic structures, and decreased risk of intraoperative complications such as ureteral damage (4–9). Laparoscopic colposuspension sec Shull (LCSS) and laparoscopic Colposacropexy (LCSP) are among the most commonly performed laparoscopic surgical interventions for POP (10, 11). LCSS is a technique that involves suspending the vaginal apex high to the uterosacral ligaments to restore the normal position of the vaginal vault and support the pelvic organs. On the other hand, LCSP entails attaching the vaginal vault to the promontories of the sacrum using a mesh, which provides robust support and solves the prolapse. Both procedures are used to resolve central compartment prolapse. Often, it is associated with anterior compartment prolapse, with varying degrees of cystocele. For this reason, cystopexy surgeries can often be combined to resolve anterior compartment defects as well. Moreover, cystopexy can also be performed to prevent stress incontinence that may develop after surgery for POP (12–16). Despite the efficacy of both procedures with or without the addition of cystopexy, comparative studies on outcomes of these two approaches exploring long-term durability of repairs, recurrence rates and complications remain underexplored. The aim of the present study is to fill this gap by comparing LCSS and LCSP without cystopexy to LCSS and LCSP with cystopexy to provide comprehensive insights on the postoperative outcomes that will guide the surgeon on clinical decision-making and will help to optimize patient care in the management of pelvic organ prolapse.

## Materials and methods

The present study is a retrospective case-control analysis carried out on women surgically treated for grade 3–4 POP-Q Uterine prolapse at the Academic Department of Gynaecology and Obstetrics of Azienda Ospedaliera Policlinico Universitario “G. Martino” of Messina, Italy, and at Azienda Ospedaliera Universitaria “L. Vanvitelli” of Napoli, Italy, between November 2020 and February 2022. Patients' data were retrieved from hospital medical records. All the patients were treated with laparoscopic hysterectomy followed by laparoscopic colpo-sacropexis (LCSP) or high uterosacral ligament suspension (LCSS) without other prolapse correction (Group A) and LCSP/LCSS associated with cystopexy for anterior defect (Group B). Urodynamic tests eventually confirmed concomitant urinary tract symptoms. All patients underwent a total hysterectomy in accordance with the clinical protocols of the recruiting centers. Additionally, all enrolled patients underwent urodynamic testing within 60 days prior to surgery.

In accord with the clinical practice of the institutions involved, cystopexy has been associated in cases of grade 2 or higher median cystocele with symptomatic repercussions by the patient or alterations reported on urodynamic tests. All included patients were treated surgically by 3 surgeons: CR, MT, and SC. All included surgeons have more than 5 years' experience in both vaginal and laparoscopic surgery for urodynamic correction. All patients are managed according to standardized clinical protocols with bladder catheter removal 48 h after surgery, bladder physiotherapy for the next 12 h, and discharge without complications on the third postoperative day. No antibiotic therapy in addition to preoperative prophylaxis is performed in the absence of complications. The timing of follow-up visits was set at 1, 3, 6, 12, and 24 months after surgery. Any complication in the first 30 days after surgery was recorded and classified according to the Clavien-Dindo classification (17). Also, prolapse recurrences were taken under control during Follow-ups.

Recurrence of anatomical prolapse was defined as any compartment descent stage II according to the POP-Q system (18).

### Technique

The LCSP and LCSS techniques were performed laparoscopically with 3 standard 5-mm accesses in the pelvis and 10-mm transumbilical optics. All patients under study underwent hysterectomy prior to colposuspension following the steps described by Gueli Alletti et al. (11). LCSP was performed according to the steps described by Henniger et al. in 2015 (19, 20), and LCSS followed the steps described by Restaino et al. in 2016 (21). Cystopexy was, on the other hand, performed vaginally with colpoincision and raffia of the bladder fascia with natural vaginal tissue repair technique (NVTR) (22).

### Statistical analysis

The primary endpoint was the rate of post-operative complications in the first 30 days. The null hypothesis (H0) of the primary endpoint of the study is devised as no difference in the prevalence of post-operative complications in the first 30 days in patients undergoing laparoscopic hysterectomy for uterine prolapse with colposuspension, compared to the same technique combined with cystopexy, (H0: π-π1 = 0; one-way). To test H0, the significance level α was set = 0.05. Fisher's exact test and Chi- squared test were used to calculate *p* in comparing dichotomous variables and Wilcoxon's test in the case of ordinal variables. Rejection of H0 for *p* ≤ α. The parameter of the presence or absence of postoperative complications in the first 30 days was estimated as a dichotomous prevalence variable. The effect of the addition of cystopexy on postoperative complications was expressed as an Odds Ratio (OR) with a 95% confidence interval. Quantitative variables such as age and BMI were expressed as medians. R software version 4.2.3 was used for statistical analysis.

### Handling of missing data and sensitivity analysis

Missing data were addressed using multiple imputation techniques, ensuring the preservation of statistical power and reducing potential bias. The imputed values were cross-validated using complete case analysis to verify consistency. Sensitivity analyses involved excluding extreme values, testing alternative statistical models, and assessing subgroup-specific trends.

### Ethical or institutional review board approval

The study was conducted in two university clinics where all patients treated must sign a dedicated consent for anonymous data processing. According to the regulations in force in the state where the study was conducted, no IRB is required due to the study's retrospective nature.

## Results

148 patients were included in the study and divided into two groups: 125 women who underwent Laparoscopic hysterectomy plus laparoscopic colposuspension (LCSP/LCSS) were included in Group A; 23 patients who were treated with the same technique with the addition of cystopexy formed Group B. Table 1 describes the main characteristics of the groups.

**Table 1:** Patients characteristics.

Characteristic	Group A,	Group B,	*p*-value^b^
	*N*^a^ = 125	*N* = 23	
Parity ≥ 2	29, (74%)	17, (68%)	0.6
Missing	84	0	
Age	59, (55.4)	60, (58.7)	0.8
BMI	26.5, (6.4)	24.9, (3.8)	0.7
Missing	88	13	
LCSP	89 (71.2)	5 (21.7)	**<0**.**001**
LCSS	35 (28.8)	18 (88.3)	
Stamey Score			0.6
0	5, (31%)	3, (16%)	
1	7, (44%)	9, (47%)	
2	4, (25%)	7, (37%)	
Missing	107	6	
Previous Surgery	55, (45%)	11, (44%)	>0.9
Previous Laparotomy	0, (0%)	3, (12%)	**0**.**004**

^a^ *n*, (%); Median, (IQR).

^b^ Pearson's Chi-squared test; Wilcoxon rank sum test; Fisher's exact test.

Bold values means the statistically significant.

The two groups were homogeneous regarding parity, BMI, Stamey Score, Age and prevalence of previous surgery. Data concerning previous laparotomy showed a statistical difference (12% Group B vs. 0% Group A; *p* = 0.004) in favor of Group B. Moreover, LCSP was statistically more frequent in Group A (71.2% vs. 21.7%; *p* < 0.001).

### Outcomes

Operative time was comparable in the two groups (*p* = 0.042): mean surgery length is 153 min in LCSP or LCSS (data concerning 84 procedures are missing) and 126 min in colposuspension associated with cystopexy. The primary endpoint was the evaluation of post-operative complications. The post-operative complications rate in Group A was 2.4% compared to 16% in Group B (*p* = 0.016). Only 3 complications occurred in Group A and 4 in Group B. Adding cystopexy at the time of colposuspension resulted in a statistically significant Odds Ratio of 7.62 (95% CI 1.59–36.51; *p* = 0.017) to develop post-operative complications in the early 30 days. The secondary outcome was the recurrence rate of prolapse at 24 months. Group A presented a recurrence rate of 13% compared to 12% of Group B (*p* > 0.9). Surgical Outcomes are summarized in Table 2.

**Table 2:** Outcomes.

Characteristic	Group A,	Group B,	*p*-value^b^
	*N*^a^ = 125	*N* = 23	
Post-Operative Complication	3, (2.4%)	4, (16%)	**0** **.** **016**
Operative Time	153, (57)	126, (52)	0.042
Missing	84	0	
Recurrences	5, (13%)	3, (12%)	>0.9
Missing	85	0	

^a^ *n*, (%); Mean, (SD).

^b^ Fisher's exact test; Wilcoxon rank sum test.

Bold values means the statistically significant.

The types of complications are summarized in Table 3.

**Table 3:** Type of complication.

Post-Operative Complication	Group A,	Group B,
	*N* = 3	*N* = 4
Anaemia with Trasfusion	0	3, (75%)
Pulmunary Embolia	1, (33%)	0
Fever	2, (66%)	1, (25%)

As reported in the previous paragraph, group A reported 3 complications: 1 case (33%) of pulmonary embolism and 2 cases (66%) of fever. On the other hand, group B reported 4 cases of postoperative complications: 3 patients had post-operative anemia with the necessity of blood transfusion (75%), and 1 patient had a post-operative fever (25%).

## Discussion

### Main findings

The present retrospective study demonstrates that adding cystopexy to LCSP or LCSS at the time of uterine prolapse treatment increases on 7 times the risk of postoperative complications in the early 30 days after surgery. The operation time seems to be slightly longer in group A (without cystopexy). However, the results of this outcome are weakened by a high presence of missing data and also could be attributed to the higher percentage of LCSP in group A. Moreover, cystopexy was not found to decrease the rate of prolapse recurrence when combined with LCSP or LCSS.

### Interpretation of results and clinical significance

Despite the common use of cystopexy, there are only a few studies on its precise indications and post- operative complication rates.

As already demonstrated, the key advantage of the laparoscopic approach for POP treatment is the magnified view of the operative field, which facilitates easier dissection and a more precise suture placement, thus helping minimize potential damage to the ureter. These benefits align with existing literature demonstrating minimal ureteric injury rates associated with this laparoscopic procedure (6, 7, 23, 24).

Otherwise, it is used in continent women at the time of surgical correction of POP to prevent genuine stress after corrective surgery for urogenital prolapse (24). In particular, Fianu et al. showed that 16% of their patients developed stress incontinence following anterior colporrhaphy (14), and Stanton et al. (13) reported an incidence of 11%. In contrast, Colombo et al. (25) observed an overall incidence of 8% of significant stress incontinence that required further treatment in two cases. The underlying causes of genuine stress incontinence following prolapse surgery remain not fully understood. In the literature (13), no differences have been found in age, parity, weight, and past pelvic surgery between the subjects who remained continent postoperatively and those who did not. In addition, it has been shown that the addition of hysterectomy does not affect postoperative outcomes in the case of surgery for urodynamic correction, as long as the surgery is not related to other clinical conditions that may alter pelvic structures (26–28). Postoperative bladder neck descent because of ineffective urethorvescical junction stabilization, and/or periuretral fibrosis with urethral denervation seem to be direct consequences of stress incontinence after POP surgery (13, 29). Our research group has previously shown that corrective surgery with a purely laparoscopic approach has an increased risk of symptom recurrence and organ prolapse (30). We believe that the occurrence of stress incontinence after prolapse surgery may have a multifactorial origin. For this reason, although the preventive execution of cystopexy could play a fundamental role in reducing this undesirable outcome, it must be remembered that cystopexy is not free from potential postoperative complications. Therefore, a careful selection of patients who could genuinely benefit from this procedure should be conducted to avoid adding unnecessary risks.

### Strengths and limitations

A major strength of the present study is its focus on cystopexy and its association with postoperative outcomes. Indeed, there are only a few studies in the literature regarding cystopexy, and most are outdated or in non-English language (23). Moreover, the cystopexy technique is never described in a standardized way. Nevertheless, all patients reported in the study underwent the same cystopexy methods by the Natural Vaginal Tissue repair technique. Hence, we believe that our work, besides presenting new data on the increased postoperative complications in patients undergoing cystopexy, also plays a fundamental role in highlighting the need for further studies helping in a better selection of patients.

A limitation of the present study is its retrospective nature, which limits the ability to establish causality and resulted in an abundance of “missing data” in the sample characteristics. This may limit the reproducibility of the study and its translation into clinical practice. Moreover, we have no data on post-surgical stress incontinence in the two groups with/without cystopexy, which is usually one of the main indications for cystopexy after surgery for POP (23). Hence, we would expect results in favor of adding the procedure routinely to prevent this unpleasant outcome. Moreover, since POP significantly impacts patients' quality of life, data on subjective patient satisfaction after surgical correction of POP would be needed, but they are still missing. Finally, laparoscopic and vaginal techniques involve two different skills, which may not coexist in the same operator (4, 31).

Therefore, the higher complication rate might be operator-dependent. Designs of prospective studies may clarify these doubts.

## Conclusions

In conclusion, it seems that patients undergoing cystopexy after surgical correction of POP are at increased risk of serious post-operative complications. However, there are no data concerning patient satisfaction and the occurrence of post-surgical stress incontinence. Hence, further studies are surely needed to better select patients who may truly benefit from cystopexy after surgical correction of POP.

## Data Availability

All data and the methodological process for their calculation can be supplied under explicit request to the corresponding author and provided as an “.R” file.
